# Granzyme A Produced by γ_9_δ_2_ T Cells Induces Human Macrophages to Inhibit Growth of an Intracellular Pathogen

**DOI:** 10.1371/journal.ppat.1003119

**Published:** 2013-01-10

**Authors:** Charles T. Spencer, Getahun Abate, Isaac G. Sakala, Mei Xia, Steven M. Truscott, Christopher S. Eickhoff, Rebecca Linn, Azra Blazevic, Sunil S. Metkar, Guangyong Peng, Christopher J. Froelich, Daniel F. Hoft

**Affiliations:** 1 Division of Infectious Diseases, Allergy and Immunology, Department of Internal Medicine, Saint Louis University, St. Louis, Missouri, United States of America; 2 Department of Molecular Microbiology and Immunology, Saint Louis University, St. Louis, Missouri, United States of America; 3 NorthShore University HealthSystems Research Institute, Evanston, Illinois, United States of America; Portland VA Medical Center/Oregon Health and Science University, United States of America

## Abstract

Human γ_9_δ_2_ T cells potently inhibit pathogenic microbes, including intracellular mycobacteria, but the key inhibitory mechanism(s) involved have not been identified. We report a novel mechanism involving the inhibition of intracellular mycobacteria by soluble granzyme A. γ_9_δ_2_ T cells produced soluble factors that could pass through 0.45 µm membranes and inhibit intracellular mycobacteria in human monocytes cultured below transwell inserts. Neutralization of TNF-α in co-cultures of infected monocytes and γ_9_δ_2_ T cells prevented inhibition, suggesting that TNF-α was the critical inhibitory factor produced by γ_9_δ_2_ T cells. However, only siRNA- mediated knockdown of TNF-α in infected monocytes, but not in γ_9_δ_2_ T cells, prevented mycobacterial growth inhibition. Investigations of other soluble factors produced by γ_9_δ_2_ T cells identified a highly significant correlation between the levels of granzyme A produced and intracellular mycobacterial growth inhibition. Furthermore, purified granzyme A alone induced inhibition of intracellular mycobacteria, while knockdown of granzyme A in γ_9_δ_2_ T cell clones blocked their inhibitory effects. The inhibitory mechanism was independent of autophagy, apoptosis, nitric oxide production, type I interferons, Fas/FasL and perforin. These results demonstrate a novel microbial defense mechanism involving granzyme A-mediated triggering of TNF-α production by monocytes leading to intracellular mycobacterial growth suppression. This pathway may provide a protective mechanism relevant for the development of new vaccines and/or immunotherapies for macrophage-resident chronic microbial infections.

## Author Summary

A small subset of human T cells express γ_9_δ_2_ T cell receptors and recognize unique non-peptide phosphoantigens expressed by microbes and damaged cells, such as cancer. These cells are important because: 1) they reside within skin and mucosal surfaces at critical points of initial pathogen invasion, and 2) they are not restricted by polymorphic HLA types and thus can be activated by the same cognate antigens in highly diverse populations. Many important human pathogens such as the causes of AIDS, malaria, tuberculosis and others induce potent responses in γ_9_δ_2_ T cells that can be protective. However, the key mechanisms involved in γ_9_δ_2_ T cell-mediated protective immunity are not well defined. We have found that γ_9_δ_2_ T cells produce soluble granzyme A which correlates with their ability to protect against intracellular mycobacterial growth. We show directly that highly purified granzyme A alone can trigger human monocytes to control intracellular mycobacteria. We further show that the granzyme A-induced mycobacterial inhibition required production of TNF-α by infected monocytes. These studies may have important implications for future vaccine development and novel therapeutic strategies.

## Introduction

While γ_9_δ_2_ T cells comprise only 3% to 5% of total human blood lymphocytes they can expand to high frequencies *in vivo* and develop effector functions protective against multiple major human pathogens [1], [2]. In addition, γ_9_δ_2_ T cells can be stimulated by non-peptidic phosphoantigens, potentially broadening the host capacity for immune recognition of invading pathogens [3]. Furthermore, γ_9_δ_2_ T cells can develop memory responses critical for long-term vaccine-induced protective immunity [4]–[6]. Although phosphoantigens (e.g.-isopentenyl pyrophosphate) can induce polyclonal γ_9_δ_2_ T cell activation and anti-tumor immunity, we have shown that these same activation conditions do not generate γ_9_δ_2_ T cells capable of inhibiting intracellular mycobacterial growth [7]. Thus, the relevant TB antigens inducing protective γ_9_δ_2_ T cells so far remain undefined. However, despite the failure of simple phosphoantigens to induce mycobacteria-inhibitory γ_9_δ_2_ T cells, effector memory γ_9_δ_2_ T cells expanded with live BCG or mycobacterial lysates can display potent effector functions relevant for protective TB immunity including production of Th1 cytokines and cytolytic activity after co-culture with mycobacteria-infected human monocytes [7]–[10]. The specific effector mechanisms most important for γ_9_δ_2_ T cell-mediated suppression of intracellular mycobacterial growth in vivo are not known.

In the present work, we have identified a novel granzyme A-dependent, but perforin-independent mechanism utilized by mycobacteria-specific γ_9_δ_2_ T cells to inhibit the growth of intracellular mycobacteria. Co-culture of infected macrophages with either mycobacteria-specific γ_9_δ_2_ T cells or purified granzyme A resulted in the production of TNF-α by target macrophages and inhibition of intracellular mycobacterial growth without inducing apoptosis. These findings are consistent with recently published data demonstrating that soluble granzyme A can trigger the production of inflammatory cytokines, including TNF-α, by macrophages without inducing apoptosis [11]. However, our results are novel by showing for the first time that the inflammatory responses induced by granzyme A can lead to intracellular anti-microbial activity. Overall, these combined results indicate that granzyme A produced by γ_9_δ_2_ T cells can induce infected macrophages to produce TNF-α which by itself and/or in combination with other factors leads to intracellular control of mycobacterial replication. These results may provide an important new target for future vaccine development and/or lead to novel therapeutic strategies for combating chronic macrophage-resident microbial infections.

## Materials and Methods

### Human samples

PPD positive, HIV negative healthy volunteers were recruited following a Saint Louis University IRB approved protocol and consent process conducted according to the principles expressed in the Declaration of Helsinki. Written informed consent was provided by all study participants. Ficoll-Paque (GE Healthcare, Piscataway, NJ) was used to obtain PBMC from leukapheresed samples. Adherent monocytes were used for the generation of mature DC as previously described [12]. γ_9_δ_2_T cell lines were generated from primary BCG-expanded γ_9_δ_2_ T cells [7]. Antigen expanded γ_9_δ_2_ T cells were purified on day 7 with immunomagnetic beads and restimulated with BCG-infected macrophages (1∶1.2 ratio) then expanded by replenishing medium with 5 U/mL rhIL-2 every 3–4 days as needed. This stimulation/expansion protocol was repeated every 2 weeks for continued maintenance of the γ_9_δ_2_ T cell lines.

### Reagents

IL-2 (Hoffmann-LaRoche Inc., Basel, Switzerland) was used to expand γ_9_δ_2_ T cell lines. Strontium chloride (Sigma Aldrich, St. Louis, MO) pre-treatment was used to induce degranulation [13]. Wortmannin and 3-Methyladenine (Sigma) were used to block autophagy [14], [15]. zVAD.fmk (BD Biosciences) was used to inhibit apoptosis [16]. The following neutralizing antibodies were used to block T cell effector functions: anti-FasL (clone NOK-1) and anti-TNF-α (clone MAb1) from BD Bioscience; anti-IFN-γ (clone 25718) from R&D Systems; and anti-Perforin (clone δG9) from Axxora, San Diego, CA. All antibodies used for flow cytometry were from BD Bioscience except anti-Granulysin from eBioscience (San Diego, CA). CFSE was from Molecular Probes (Eugene, OR). Phorbol myristate acetate (PMA, Sigma-Aldrich), ionomycin (Sigma-Aldrich) and Cytofix/Cytoperm kit (BD Biosciences) were used in the intracellular staining protocol. Soluble 4-hydroxy-but-2-enyl pyrophosphate (HMB-PP, Echelon Bioscience, Salt Lake City, UT) was used for stimulation of γ_9_δ_2_ T cells in some transwell conditions. Granzyme A was quantified with the Human Granzyme A CBA Flex Set (BD Biosciences), collected on a LSR II flow cytometer and analyzed with FCAP Array software (BD Biosciences). Native human granzyme A was isolated from NK92 cells, quantified by silver stain, shown not to be contaminated with granzyme B or M, and endotoxin depleted as previously described [17]. Briefly, the postnuclear fraction of centrifuged disrupted NK92 cells was spun at 14,500×g for 15 min to obtain the granule pellet. This was extracted 3 times by 0.5% Triton X-100 and each fraction subjected to cation-exchange chromatography. All gradient fractions were individually assayed for protein concentration and esterolytic activity. No contaminating proteins were observed by silver staining of the final native granzyme A preparation. In addition, the final native granzyme A preparation was shown to be free of granzyme B and M by Western blot. Any endotoxin contamination of purified granzyme A was removed using the EndoTrap-Blue kit (Cambrex Bioscience Walkersville Inc, Walkersville, MD). Protein concentration of the endotoxin free granzyme was determined by Coomassie stain. The efficiency of the EndoTrap-Blue kit was verified by applying LPS to the EndoTrap column examining the flow-through for residual endotoxin activity using the Kinetic QCL kit (Cambrex Bioscience Walkersville Inc,). Efficiency of endotoxin depletion was shown to be >98%.

### Preparation of and monocyte infection by mycobacteria

BCG was grown in Middlebrook 7H9 media supplemented with 10% albumin, dextrose, catalase (ADC)+0.05% tween. Aliquoted stocks were frozen and the concentration determined after thawing. Thawed aliquots were sonicated to generate single-cell suspensions before dilution and infection of monocytes. *Mycobacterium tuberculosis* H37Rv (Mtb, from BEI Resources, Manassas, VA) was grown in Middlebrook 7H9 media containing ADC enrichment and 0.1% glycerol. Aliquoted stocks were frozen and the concentration determined after thawing. Thawed aliquots were vortexed for 5 minutes to disrupt clumps before dilution and infection of monocytes in the presence of 200 nM granzyme A. Plastic adherent PBMCs were either used fresh or differentiated for 6 days before infection with a mycobacterial multiplicity of infection (MOI) of 3 as previously described [18]. Following removal of extracellular mycobacteria, infected monocytes were either cultured in medium alone or with γ_9_δ_2_ T cells (E∶T 10∶1) or soluble granzyme A at the indicated concentrations.

### Assay of γδ T cell-mediated inhibition of intracellular mycobacterial growth

Two densities of adherent monocyte targets (∼1.5×10^4^ and ∼1.2×10^5^/well) were used as previously described [18]. Briefly, BCG- or Mtb-infected (MOI = 3) monocytes were cultured for 3 days with γ_9_δ_2_ T cell lines at an effector to target ratio of 10∶1. In assays with lower density of monocytes, medium-rested PBMC (1.5×10^5^/well) were added to support optimal intracellular mycobacterial growth as previously described [18]. Residual intracellular BCG were measured by incorporation of H^3^-uridine and colony forming unit (CFU) counting on 7H10 Middlebrook agar plates. Percentages of mycobacterial growth inhibition were determined as follows: % inhibition = 100 - [100×(CFU or DPM in the presence of γ_9_δ_2_ T cells/CFU or disintegrations per minute (DPM) in the absence of γ_9_δ_2_ T cells)]. In transwell experiments, additional BCG-infected macrophages (∼4×10^4^) were seeded in the upper chambers of the transwell inserts (0.4 µm HTS-96 from Corning, Acton, MA or 8 well strips from Nunc, Rochester, NY). γ_9_δ_2_ T cells were added at a 10∶1 E∶T ratio either to the top or bottom layers of infected monocytes/macrophages and the bottom layers harvested 3 days later for viable BCG/Mtb quantification.

### Construction and use of lentivirus expressing shRNA for knockdown experiments

siRNAs were purchased from Applied Biosystems (Foster City, CA) targeting TNF-α, IFN-γ, perforin, and GAPDH, and cloned into the pSilencer adeno 1.0 CMV shuttle plasmid (Applied Biosystems). To evaluate effectiveness of siRNA-induced knockdown, plasmid DNA was transfected into THP-1 monocytes (ATCC, Manassas, MO) using Nucleofection (Amaxxa, Walkersville, MD) (Fig. S1). Shuttle vectors and adenovirus backbone were co-transfected into 293T cells and viral particles purified using the Adeno-X purification system (Clontech, Mountain View, CA). Viral titers were determined in 293T cells. γ_9_δ_2_ T cells or macrophages were infected with siRNA-expressing adenovirus for 4 hours 1 day prior to co-culture. For silencing of Granzyme A, three siRNA sequences for human GzmA (NM_006144.3) were selected with use of computer-assisted programs. Oligonucleotides containing a siRNA sequence, 8 nucleotide spacers, and a polyT terminator sequence were annealed and then cloned into the HapI and XhoI sites of GFP-expressing pLentilox3.7 vector [19]. siRNA was under the control of a U6 promoter. After screening, only one siRNA generating construct (GzmA2-siRNA: GATTGGAATGAATATGGTTTG) effectively silenced GzmA expression in γ_9_δ_2_ T cells. 293T cells were transfected with 13 µg siRNA lentiviral DNA [19], 6 µg VSV-G plasmid DNA [19] and 9 µg packaging viral CMV delta 8.9 plasmid [19], for 8 hours using Lipofectamine 2000 (Invitrogen). Viral supernatants were harvested 2–3 days after transfection, filtered and centrifuged at 20,000 rpm for 2 h. Virus pellets were resuspended and viral titers were determined in 293T cells. For transduction of γ_9_δ_2_ T cells, T cells were pre-activated with OKT3 (2 µg/ml)-coated plates, mixed with lentiviral supernatant at an MOI of 20 in medium containing 8 µg/ml polybrene (Sigma), and centrifuged at 1100×g for 1 h at room temperature. Fresh medium was added to each well after 16 h and transduction was repeated two days later. Transduction efficiency was analyzed 3–4 days post-transduction (Fig. S5). Transduced cells and control T cells were then used in the mycobacterial growth inhibition assay.

### Statistical analysis

Microsoft Excel, GraphPad Prism or StatSoft Statistica were used for analysis. Mann-Whitney U, ANOVA Friedman test with Dunn's multiple comparisons test or Wilcoxon matched-pairs tests were used as appropriate.

## Results

### Direct inhibition of intracellular mycobacterial growth by mycobacteria-specific γ_9_δ_2_ T cells is dependent on soluble factors including TNF-α

**Figure 1:**
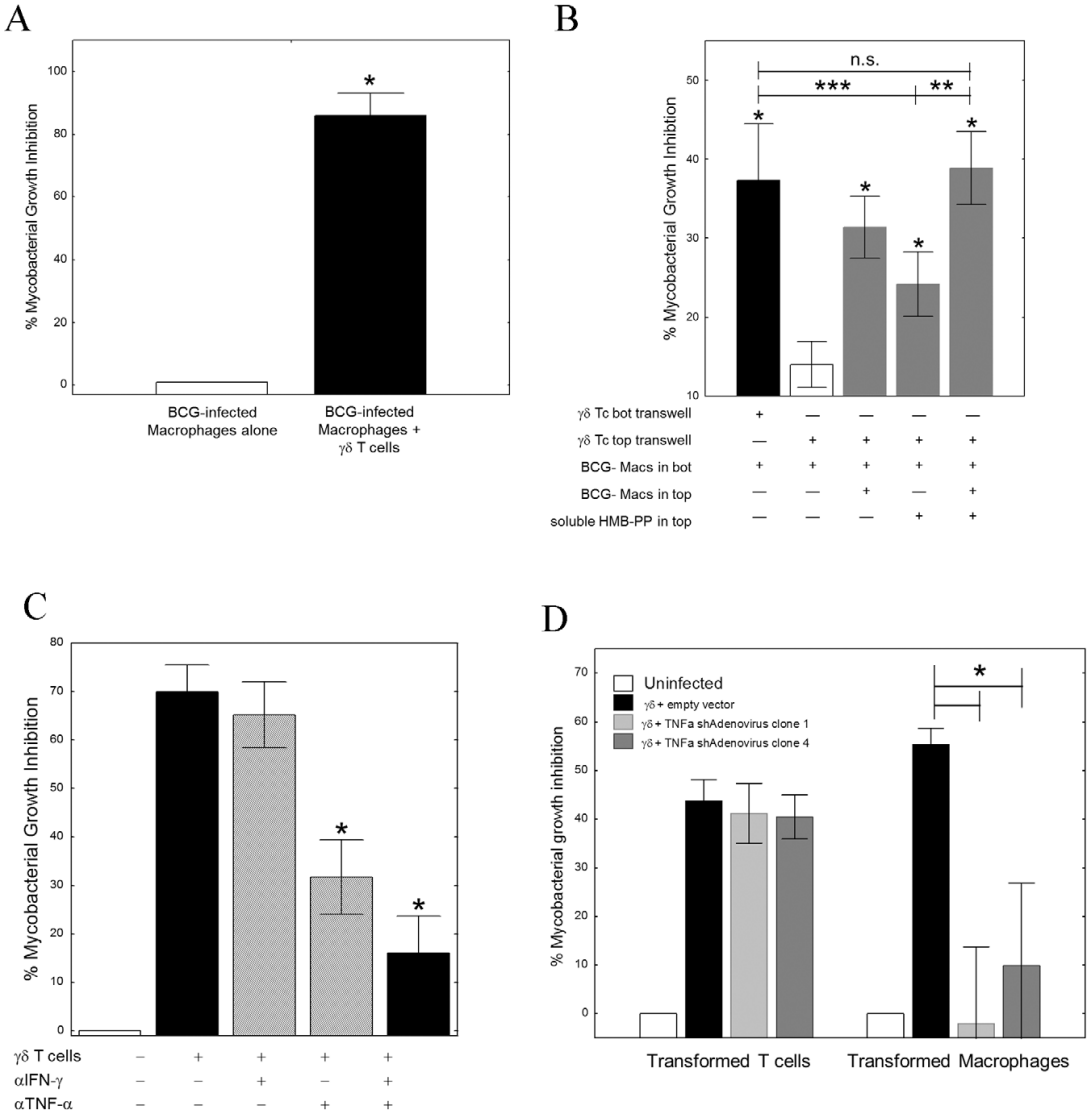
TNF-α is critical for γ_9_δ_2_ T cell-mediated inhibition of intracellular mycobacterial growth. *A*, γ_9_δ_2_ T cell lines significantly suppressed intracellular growth of mycobacteria (n = 5; *p<0.05 by Wilcoxon matched-pairs test). γ_9_δ_2_ T cells were co-cultured with BCG-infected macrophages for 3 d. Surviving bacteria were quantified by H^3^-uridine incorporation and compared with cultures absent of γ_9_δ_2_ T cells. *B*, The effector mechanism responsible for γ_9_δ_2_ T cell-mediated mycobacterial growth inhibition involves soluble factors. γ_9_δ_2_ T cells were stimulated in the top chambers of semi-permeable transwell membrane (0.4 µm pores) systems with BCG-infected macrophages and/or soluble HMB-PP. The levels of intracellular growth inhibition were assayed in the bottom chambers and compared to the levels of inhibition achieved by direct co-culture of γ_9_δ_2_ T cells with the bottom layer of BCG-infected macrophages (*p<0.05, n = 5 by Wilcoxon matched pairs tests compared with unstimulated γ_9_δ_2_ T cells separated by transwells; **p<0.02, n = 7 by Wilcoxon matched pairs tests compared with HMB-PP stimulated γδ T cells separated by transwells; ***p<0.003, n = 15 by Wilcoxon matched pairs tests compared with HMB-PP stimulated γδ T cells separated by transwells). *C*, Neutralization of TNF-α alone prevents γ_9_δ_2_ T cell-mediated inhibition while neutralization of IFN-γ alone did not (*p<0.02, n = 7 by Wilcoxon matched pairs tests compared with no antibody control; **p<0.02, n = 6 by Wilcoxon matched pairs tests compared with no antibody control; n.s. – not significantly different). γ_9_δ_2_ T cells were co-cultured with BCG-infected macrophages in the presence or absence of the indicated neutralizing antibody and the surviving bacteria quantified by H^3^-uridine incorporation. *D*, shRNA-mediated knockdown of TNF-α in macrophages, but not γ_9_δ_2_ T cells, eliminates γ_9_δ_2_ T cell-mediated mycobacterial growth inhibition. (*p<0.02, n = 7 by Wilcoxon matched pairs tests compared with adenovirus expressing the empty vector). γ_9_δ_2_ T cells were co-cultured with BCG-infected macrophages. Prior to co-culture, either the T cells or uninfected macrophages were infected with shRNA expressing Adenovirus. Surviving bacteria were quantified by H^3^-uridine incorporation.

Addition of BCG-specific γ_9_δ_2_ T cell lines or clones (data not shown) to BCG-infected macrophages resulted in intracellular mycobacterial growth inhibition (Fig. 1*A*; *p<0.05 by Wilcoxon matched pairs test, n = 5), as previously reported [7]. This inhibition did not require cell-to-cell contact as activated BCG-specific γ_9_δ_2_ T cells in the upper transwell chambers could suppress intracellular mycobacterial growth in monocytes plated in the lower chambers. However, stimulation with both BCG-infected macrophages and soluble 4-hydroxy-but-2-enyl pyrophosphate (HMB-PP) in the upper transwell chambers resulted in maximal inhibition of mycobacterial growth in the lower chambers (Fig. 1*B*), probably due to optimal production of soluble inhibitory factors mediating inhibitory effects. These combined results show that the effector mechanism that inhibits intracellular mycobacteria does not require contact between the γ_9_δ_2_ T cells producing the soluble effector molecules and the target infected macrophages, although the triggering of the protective response is contact dependent. Effector molecules produced by γ_9_δ_2_ T cells placed above the transwell inserts can pass through the membrane and induce macrophages placed below the membrane to better control intracellular mycobacterial growth. However, the γ_9_δ_2_ T cells above the membrane must be stimulated by cell contact with APCs presenting BCG antigens or HMBPP to produce the soluble effector molecules that can pass through the membrane and induce the enhanced control of intracellular mycobacteria in macrophages below the membrane. In any case, it is clear that soluble molecules secreted by activated γ_9_δ_2_ T cells can inhibit the survival of intracellular mycobacteria.

IFN-γ and TNF-α have previously been implicated in the control of mycobacterial infections. Therefore, we next studied the effects of neutralization of these cytokines on γ_9_δ_2_ T cell-mediated inhibition of intracellular mycobacteria. Neutralization of IFN-γ alone did not affect mycobacterial growth inhibition. In contrast, neutralization of TNF-α significantly reduced γ_9_δ_2_ T cell-mediated inhibitory effects (Fig. 1*C*, S2; *p<0.03 by Wilcoxon matched pairs test, n = 7). These results demonstrate that TNF-α is critical for suppression of mycobacterial growth, consistent with the observed clinical effects of TNF-α blockade increasing the risks of TB disease reactivation. However, we were surprised to find in siRNA knockdown experiments that only the production of TNF-α by BCG-infected macrophages and not by γ_9_δ_2_ T cells was required for inhibition of intracellular mycobacterial growth (Fig. 1*D*; *p = 0.018 by Wilcoxon matched pairs test for both siRNA sequences, n = 7). Further studies indicated that autophagy, apoptosis, cytolysis, IFN-β and nitric oxide production were not involved in γ_9_δ_2_ T cell-mediated inhibition of intracellular mycobacteria (Fig. S3, S4). Our combined results indicate that inhibitory γ_9_δ_2_ T cells produce soluble factors that by some novel pathway activate BCG-infected macrophages to produce TNF-α and inhibit intracellular mycobacterial growth.

### Classical cell-to-cell contact mechanisms are not involved in γδ T cell inhibitory activity

**Figure 2:**
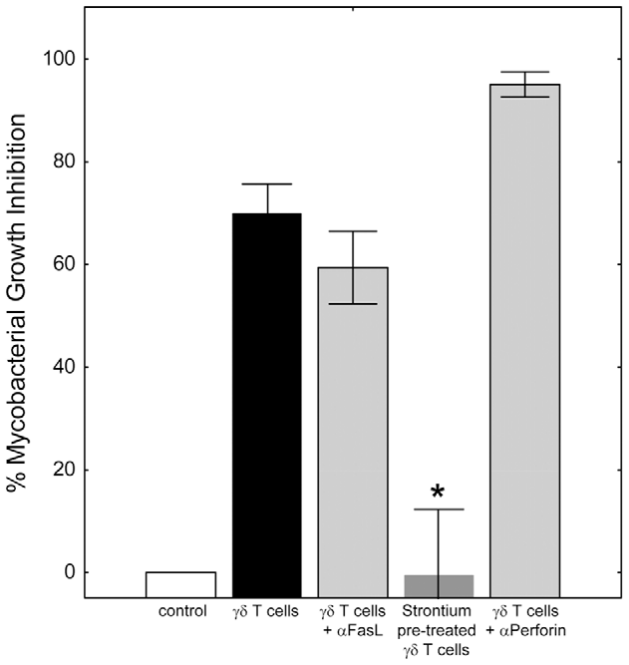
Classical effector mechanisms involving direct contact of γ_9_δ_2_ T cells with BCG-infected macrophages are not required for mycobacterial suppression. γ_9_δ_2_ T cells were co-cultured with BCG-infected macrophages in the presence or absence of the indicated treatment and surviving bacteria quantified by H^3^-uridine incorporation. Blocking of Fas/FasL interactions did not prevent γ_9_δ_2_ T cell-mediated inhibition (p = 0.23, n = 7). In contrast, γ_9_δ_2_ T cells pre-treated for 16 hours with 25 mM strontium chloride were unable to mediate mycobacterial inhibition (*p<0.05, n = 5 by Wilcoxon matched pairs test compared with untreated γ_9_δ_2_ T cells). Despite strontium depletion indicating the importance of γ_9_δ_2_ T cell cytolytic granules for mycobacterial inhibitory effects, antibody neutralization of perforin had no effect (p = 0.28, n = 3).

We considered the possibility that cell-to-cell contact mechanisms may also be involved in mycobacterial suppression. Antibody blockade of the Fas/FasL interaction did not interfere with the ability of γ_9_δ_2_ T cells to inhibit intracellular mycobacterial growth (Fig. 2; *p = 0.23 by Wilcoxon matched pairs test, n = 7). Inactivation of the cytolytic granule pathway (known to destroy target cells engaged via the immunological synapse) in γ_9_δ_2_ T cells by strontium pre-treatment, completely eliminated the capacity of these cells to suppress intracellular mycobacterial growth (*p<0.05 by Wilcoxon matched pairs test, n = 5). However, a perforin-specific neutralizing antibody previously shown to inhibit cytolysis [9] did not reverse γ_9_δ_2_ T cell-mediated mycobacterial growth suppression (p = 0.28 by Wilcoxon matched pairs test, n = 3). Overall, these results suggest that the cytolytic granule cargo secreted by activated γ_9_δ_2_ T cells contain soluble factors which stimulate infected macrophages to inhibit intracellular mycobacterial growth even in the absence of perforin.

### Mycobacteria-specific γ_9_δ_2_ T cells utilize granzyme A to trigger pro-inflammatory TNF-α

**Figure 3:**
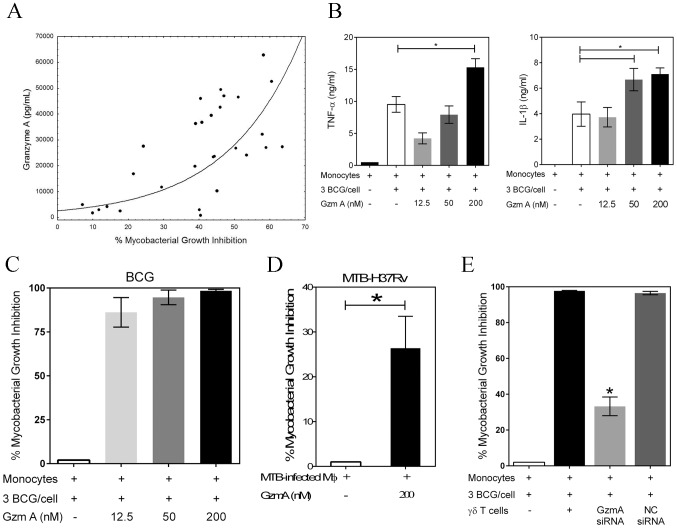
Granzyme A secretion by γ_9_δ_2_ T cells mediates inhibition of intracellular mycobacteria by induction of inflammatory responses in mycobacteria-infected macrophages. *A*, The levels of granzyme A produced by γ_9_δ_2_ T cells are directly and highly correlated with inhibition of intracellular mycobacterial growth (r = 0.7564; p<0.0001). Supernatant levels of granzyme A, measured by CBA, were correlated with the level of mycobacterial growth inhibition observed in γ_9_δ_2_ T cell co-cultures with BCG-infected macrophages. *B*, Purified granzyme A induces the production of pro-inflammatory cytokines TNF-α and IL-1β by BCG-infected macrophages (*p<0.05, n = 4; statistical comparisons performed using Friedman's test). The indicated concentrations of purified granzyme A were added to BCG-infected monocytes for 3 days and the levels of TNF-α and IL-1β in the culture supernatants determined by CBA. *C-D*, Purified granzyme A alone can induce inhibition of intracellular mycobacterial growth in the absence of perforin. The indicated concentrations of granzyme A were added to BCG-infected (*C*) or *M. tuberculosis* H37Rv-infected (*D*) co-cultures for 3 days and the surviving bacteria quantified by H^3^-uridine incorporation and CFU counting. (*p<0.03; n = 6–12 by Wilcoxon matched pairs test comparing levels of mycobacterial intracellular growth in cultures with and without added purified granzyme A). Purified granzyme A had no direct effects on extracellular mycobacterial growth (data not shown). *E*, siRNA knockdown of granzyme A in γ_9_δ_2_ T cells reduces the inhibitory activity of γ_9_δ_2_ T cells for mycobacterial growth (*p<0.05; n = 3 by one-way ANOVA comparing levels of intracellular mycobacterial growth inhibition in cultures with or without γ_9_δ_2_ T cells producing granzyme A). γ_9_δ_2_ T cells transduced with the indicated siRNA-lentivirus targeting GzmA or a negative control (NC) were co-cultured with BCG-infected macrophages for 3 days and surviving bacteria quantified by H^3^-uridine incorporation.

Granzyme A, a component of cytolytic granules, recently has been shown to induce numerous pro-inflammatory cytokines including TNF-α [11]. We hypothesized that granzyme A produced by γ_9_δ_2_ T cells (Fig. S5 *A*) may trigger macrophage TNF-α production and subsequent mycobacterial inhibition. Indeed, the levels of granzyme A in γ_9_δ_2_ T cell culture supernatants were found to be highly correlated with inhibition of intracellular mycobacteria (Fig. 3*A*; r = 0.7564, p<0.0001). In contrast, the supernatant levels of granzyme B, another component of cytolytic granules, weakly correlated with levels of inhibition (data not shown, r = 0.2298). Furthermore, purified human granzyme A induced dose-dependent increases in TNF-α and IL-1β by BCG-infected macrophages (Fig. 3*B*; p<0.05). More importantly, addition of highly purified, endotoxin-free granzyme A preparation alone to BCG-infected (Fig. 3*C*) or *M. tuberculosis* H37Rv-infected (Fig. 3*D*) macrophages resulted in highly significant inhibition of intracellular mycobacterial growth (p<0.03 by Wilcoxon matched pairs test, n = 6–12). Conversely, purified granzyme A had no direct effects on extracellular mycobacterial growth (data not shown). These data demonstrate for the first time that granzyme A alone can induce immunity protective against an intracellular pathogen.

In order to demonstrate that γ_9_δ_2_ T cells utilize this mechanism to suppress intracellular mycobacterial growth, lentiviral vectors containing shRNA constructs were generated to express siRNAs targeting granzyme A. γ_9_δ_2_ T cell lines were transduced with GzmA targeting or negative control (NC) siRNA lentivirus prior to co-culture with BCG-infected macrophages. Transduction of γ_9_δ_2_ T cells with the siRNA targeting granzyme A resulted in 50% decreases in the intracellular levels of granzyme A (Fig. S5). Associated with the GzmA knockdown was a significant reversal of the inhibitory effects of γ_9_δ_2_ for mycobacterial intracellular growth (Fig. 3*E*; p<0.05). These latter data confirm that granzyme A production represents a critical mechanism responsible for the ability of γ_9_δ_2_ T cells to inhibit intracellular mycobacterial growth.

**Figure 4:**
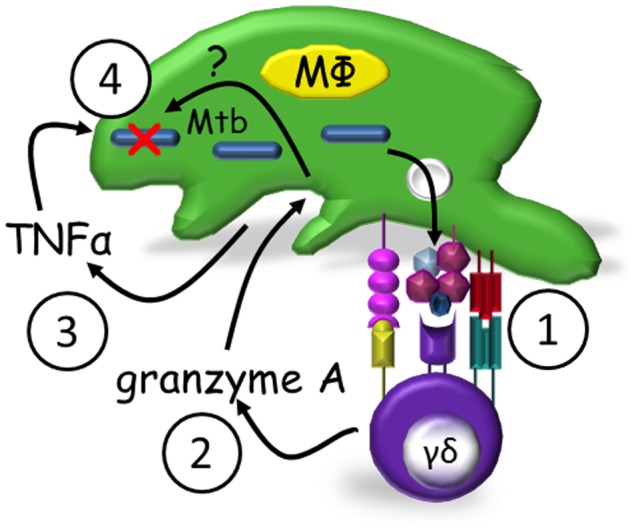
Proposed model of granzyme A-mediated suppression of intracellular mycobacterial growth by γ_9_δ_2_ T cells. Mycobacteria-infected macrophages present antigens to γ_9_δ_2_ T cells (1) which secrete granzyme A upon activation (2). Granzyme A in turn induces TNF-α production by infected and/or bystander macrophages (3) apparently independent of perforin. TNF-α activates intracellular mechanisms which alone or in concert with other unknown granzyme A-induced intracellular mechanisms suppress mycobacterial growth (4).

These data lead us to propose the following model for γ_9_δ_2_ T cell-mediated control of mycobacterial replication (Fig. 4). Mycobacteria-infected macrophages present pathogen-specific or induced self antigens to γ_9_δ_2_ T cells. Activated γ_9_δ_2_ T cells then secrete granzyme A which acts upon the macrophages to produce TNF-α. It is unclear as yet whether bystander uninfected macrophages are similarly triggered to produce TNF-α or whether TNF-α is soluble vs membrane bound. TNF-α then acts in an autocrine and/or paracrine mechanism to induce infected macrophages to control mycobacterial replication. The intracellular details by which TNF-α causes this repression require further investigation. It is also likely that intracellular molecular events in addition to TNF-α production are activated by granzyme A and contribute to the mycobacterial inhibitory effects.

## Discussion

γ_9_δ_2_ T cells have certain unique characteristics potentially important for the development of new vaccines capable of inducing protective immunity against several important human pathogens. Antigen recognition by these cells is not MHC restricted [20]. Therefore, the same antigens could induce protective γ_9_δ_2_ T cell responses in individuals from diverse genetic backgrounds. In addition, γ_9_δ_2_ T cells tend to accumulate in mucosal epithelial tissues, including the lung, where they can serve as a potent first line of defense against pathogenic microbes – e.g. mycobacteria [21], [22]. These properties make γ_9_δ_2_ T cells well-suited as early responders to infection. Furthermore, unlike conventional αβ T cells, which respond to peptide antigens, γ_9_δ_2_ T cells are activated by naturally occurring prenyl pyrophosphate metabolites (e.g.- isopentenyl pyrophosphate, IPP, and HMB-PP) [23], [24] expressed by actively replicating mycobacteria and other pathogens [3]. Thus, the stimulation of γ_9_δ_2_ T cells with prenyl pyrophosphates could represent a novel vaccine strategy. However, the effector mechanisms utilized by γ_9_δ_2_ T cells to inhibit intracellular mycobacterial replication have been unclear. Therefore, we sought to identify the key effector pathways of mycobacteria-stimulated γ_9_δ_2_ T cells that specifically act to inhibit the intracellular growth of mycobacteria.

Activated γ_9_δ_2_ T cells express numerous effector molecules including IFN-γ, TNF-α, perforin, granzyme and granulysin, all implicated in control of infectious microbes [8]–[10], [25]–[27]. Indeed, γ_9_δ_2_ T cells have been shown to be more potent producers of IFN-γ than CD4^+^ and CD8^+^ αβ T cells after re-stimulation in vitro with *M. tuberculosis*-infected APC [28], [29]. However, in addition to displaying cytotoxic activity, γ_9_δ_2_ T cells also stimulate other cell types to produce pro-inflammatory cytokines such as IFN-γ and TNF-α [30]–[33]. Our results provide further evidence indicating that γ_9_δ_2_ T cells both stimulate as well as express protective pro-inflammatory responses.

To explore the importance of soluble effector molecules in γ_9_δ_2_ T cell-mediated mycobacterial control, we neutralized the biological activity of IFN-γ, TNF-α and perforin in co-cultures of mycobacteria-protective γ_9_δ_2_ T cells and infected macrophages. Only the neutralization of TNF-α blocked the ability of mycobacteria-protective γ_9_δ_2_ T cells to control mycobacterial growth. These results indicate that TNF-α is critical for the suppression of mycobacterial growth, an observation confirmed by the clinical effects of TNF-α blockade leading to reactivation of latent TB infection. However, as demonstrated by siRNA knockdown experiments, macrophages and not γ_9_δ_2_ T cells were found to be the critical source of TNF-α for inhibition of intracellular mycobacterial growth in BCG-infected co-cultures.

The delivery of granzyme A to monocyte/macrophage cells, either as the isolated protein or by cytotoxic cells, has been shown to induce numerous pro-inflammatory cytokines including TNF-α [34]. Mycobacterial growth inhibition in our experiments strongly correlated with the levels of granzyme A in γ_9_δ_2_ T cell culture supernatants, while neutralization of perforin had no effect on mycobacterial growth inhibition. Our data support the hypothesis that granzyme A secreted by BCG-specific γ_9_δ_2_ T cells induces target macrophages to produce TNF-α which alone or in combination with unidentified factors inhibits intracellular mycobacterial growth. To our knowledge this is the first demonstration that the pro-inflammatory response elicited by granzyme A contributes to anti-pathogen host defense through microbial elimination. Furthermore, we present evidence that granzyme A triggers this protective response through a pathway that does not involve nitric oxide, type I interferons, autophagy and/or apoptosis (supplementary data).

We believe that the mechanism responsible for how granzyme A induces inhibition of intracellular mycobacteria is complex. TNF-α production by the macrophage is clearly necessary; anti-TNF neutralizing antibody prevents γ_9_δ_2_ T cell inhibitory activity and macrophage TNF knockdown prevents inhibition of intracellular mycobacterial growth. However, TNF-α is a pleiotropic cytokine contributing to both protective immunity and pathologic responses depending upon concentration and duration of production (e.g. TB reactivation occurs after TNF-α blockade, while cachexia or extreme wasting is caused by chronic excessive TNF-α production). In addition, we have shown previously [18] that high levels of TNF production by macrophages can be a marker of uncontrolled intracellular mycobacterial growth, and others have reported similar phenomena. Therefore, we believe that granzyme A-mediated induction of intracellular mycobacteria involves more than TNF-α production. TNF is required for the protective response, but there is not a simple quantitative relationship between the levels of TNF induced and the magnitude of inhibition of intracellular mycobacterial growth. We are currently trying to identify the more complete molecular signature responsible for granzyme A-mediated protective responses active against intracellular mycobacteria using a microarray approach.

Nevertheless, TNF-α production by infected or bystander macrophages may be necessary for the complete induction of γ_9_δ_2_ T cell effector functions mediating mycobacterial suppression [34]. Thus, future TB vaccine or therapeutic strategies should maximize the induction of mycobacteria-specific, granzyme A-producing γ_9_δ_2_ T cells and thereby favor the early and enhanced production of TNF-α in mycobacteria-infected and bystander macrophages in response to TB challenge.

## Supporting Information

Figure S1 **Efficacy of TNF-α knockdown by 2 small-hairpin RNA (shRNA) constructs.** THP-1 cells were transformed with plasmids encoding shRNA constructs specific for mouse TNF-α (Ambion; clone 1 and 4) by electroporation. Following overnight culture to allow degradation of TNF-α mRNA, 1 µg/mL LPS was added to the cultures in order to stimulate the secretion of TNF-α. Cytokine concentration in the supernatant was determined by ELISA 48 hours later.
(TIF)

Figure S2 **αIFN-γ (MAB285) and αTNF-α (Mab1) are able to neutralize the biological activity of purified IFN-γ and TNF-α, respectively.** The indicated cell lines were plated in 96 well plates and 10 µg/mL αTNF-α or αIFN-γ antibody added to the appropriate samples. A titration of recombinant human IFN-γ (A & B) or TNF-α (C & D) was added to stimulate the cells. The assays were cultured for 3–4 days and then pulsed with 0.5 µCi H^3^-thymidine overnight to measure the viability of the cell populations.
(TIF)

Figure S3 **TNF-α triggered by protective γ_9_δ_2_ T cells does not induce caspase-mediated apoptosis or immune-induced autophagy.** The cell-permeable general caspase inhibitor, zVAD.fmk, or autophagy inhibitors, 3-methyadenine and Wortmannin, were added to co-cultures of protective γ_9_δ_2_ T cells and infected macrophages. After 3 days of culture, the viability of intracellular mycobacteria was quantitated by H^3^-uridine incorporation.
(TIF)

Figure S4 **Protective γ_9_δ_2_ T cells do not induce IFN-β production or nitric oxide in order to inhibit intracellular mycobacterial growth.** 3 days after co-culture of BCG-infected macrophages alone or co-cultured with protective γ_9_δ_2_ T cells (left panel), soluble IFN-β was measured in culture supernatants by ELISA (middle panel) and soluble nitric oxide was measured in culture supernatants by Griess reaction (right panel).
(TIF)

Figure S5 **γ_9_δ_2_ T cells express granzyme A; the expression of which can be knockdown by siRNA-mediated inhibition.** **A**) Intracellular levels of granzyme A in γ_9_δ_2_ T cells was determined by intracellular cytokine staining. **B**) The intracellular levels of granzyme A in γ_9_δ_2_ T cells transduced with lentivirus vectors containing shRNA constructs generating granzyme A targeting or negative control (NC) siRNA was determined as in **A**. Transduced γ_9_δ_2_ T cells expressed ∼50% less granzyme A protein than did untreated γ_9_δ_2_ T cells or γ_9_δ_2_ T cells transduced with a noncoding siRNA.
(TIF)

Text S1 **Materials and Methods** **for supplemental data.** The experimental protocols used to generate data presented in the supplemental figures are reported herein.
(DOCX)
