# Supplementary figures and images for: Granzyme A Produced by γ9δ2 T Cells Induces Human Macrophages to Inhibit Growth of an Intracellular Pathogen
Source: PLoS Pathog. 2013 Jan 10;9(1):e1003119. doi: 10.1371/journal.ppat.1003119 (PMC3542113; doi:10.1371/journal.ppat.1003119)

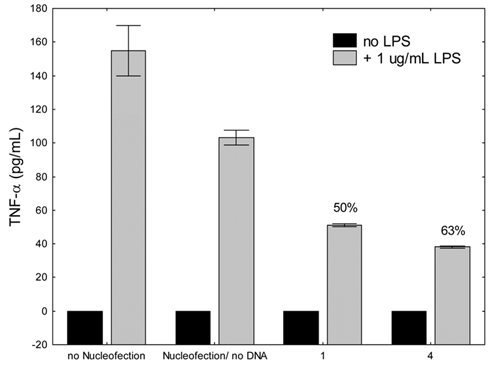

Supplement: Figure S1 — Efficacy of TNF-α knockdown by 2 small-hairpin RNA (shRNA) constructs. THP-1 cells were transformed with plasmids encoding shRNA constructs specific for mouse TNF-α (Ambion; clone 1 and 4) by electroporation. Following overnight culture to allow degradation of TNF-α mRNA, 1 µg/mL LPS was added to the cultures in order to stimulate the secretion of TNF-α. Cytokine concentration in the supernatant was determined by ELISA 48 hours later. (TIF) [file ppat.1003119.s001.tif]

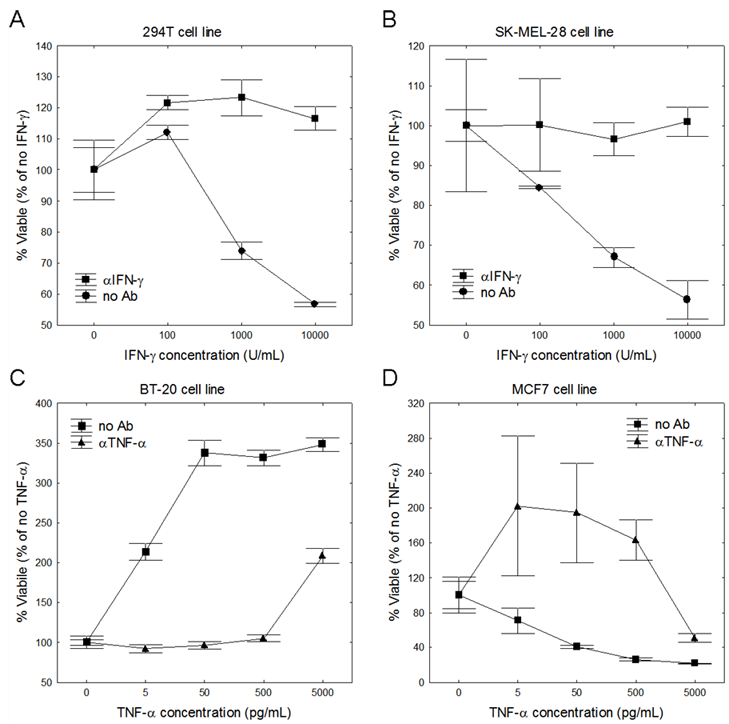

Supplement: Figure S2 — αIFN-γ (MAB285) and αTNF-α (Mab1) are able to neutralize the biological activity of purified IFN-γ and TNF-α, respectively. The indicated cell lines were plated in 96 well plates and 10 µg/mL αTNF-α or αIFN-γ antibody added to the appropriate samples. A titration of recombinant human IFN-γ (A & B) or TNF-α (C & D) was added to stimulate the cells. The assays were cultured for 3–4 days and then pulsed with 0.5 µCi H3-thymidine overnight to measure the viability of the cell populations. (TIF) [file ppat.1003119.s002.tif]

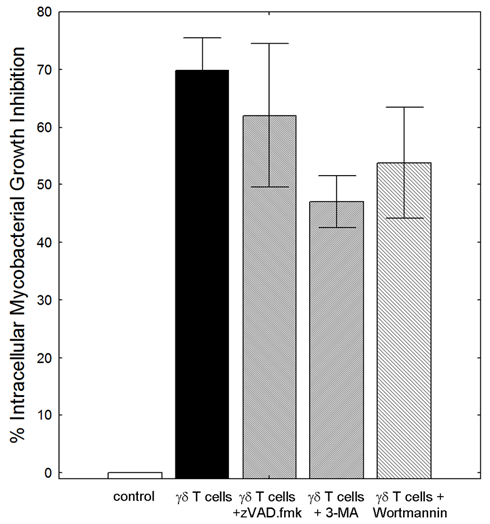

Supplement: Figure S3 — TNF-α triggered by protective γ9δ2 T cells does not induce caspase-mediated apoptosis or immune-induced autophagy. The cell-permeable general caspase inhibitor, zVAD.fmk, or autophagy inhibitors, 3-methyadenine and Wortmannin, were added to co-cultures of protective γ9δ2 T cells and infected macrophages. After 3 days of culture, the viability of intracellular mycobacteria was quantitated by H3-uridine incorporation. (TIF) [file ppat.1003119.s003.tif]

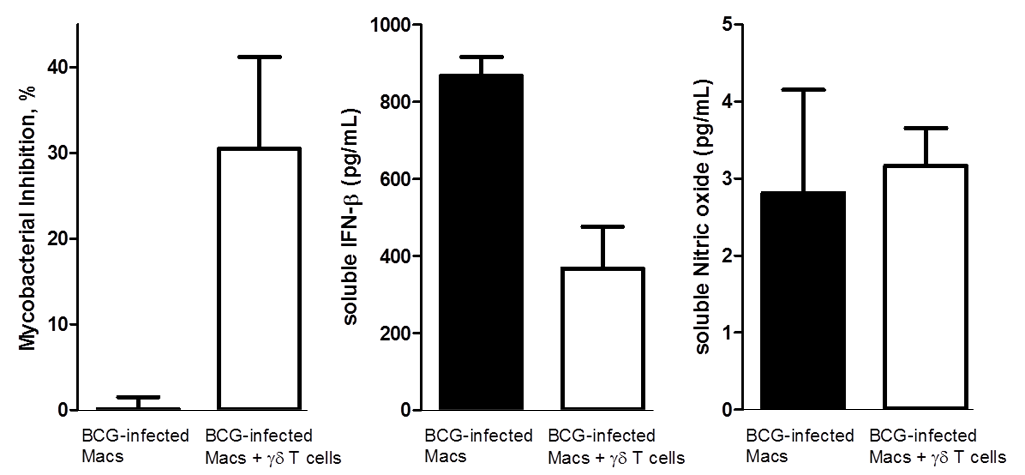

Supplement: Figure S4 — Protective γ9δ2 T cells do not induce IFN-β production or nitric oxide in order to inhibit intracellular mycobacterial growth. 3 days after co-culture of BCG-infected macrophages alone or co-cultured with protective γ9δ2 T cells (left panel), soluble IFN-β was measured in culture supernatants by ELISA (middle panel) and soluble nitric oxide was measured in culture supernatants by Griess reaction (right panel). (TIF) [file ppat.1003119.s004.tif]

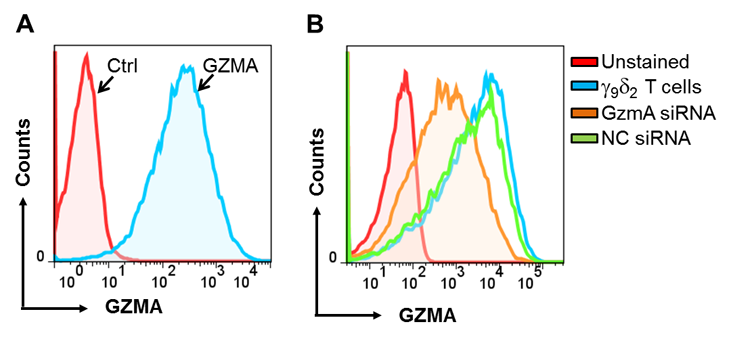

Supplement: Figure S5 — γ9δ2 T cells express granzyme A; the expression of which can be knockdown by siRNA-mediated inhibition. A) Intracellular levels of granzyme A in γ9δ2 T cells was determined by intracellular cytokine staining. B) The intracellular levels of granzyme A in γ9δ2 T cells transduced with lentivirus vectors containing shRNA constructs generating granzyme A targeting or negative control (NC) siRNA was determined as in A. Transduced γ9δ2 T cells expressed ∼50% less granzyme A protein than did untreated γ9δ2 T cells or γ9δ2 T cells transduced with a noncoding siRNA. (TIF) [file ppat.1003119.s005.tif]
