# Supplementary material for: Granzyme A Produced by γ9δ2 T Cells Induces Human Macrophages to Inhibit Growth of an Intracellular Pathogen
Source: PLoS Pathog. 2013 Jan 10;9(1):e1003119. doi: 10.1371/journal.ppat.1003119 (PMC3542113; doi:10.1371/journal.ppat.1003119)
Supplement: Text S1 — Materials and Methods for supplemental data. The experimental protocols used to generate data presented in the supplemental figures are reported herein. (DOCX) [file ppat.1003119.s006.docx]

**Supporting Materials and Methods**

**Verification of TNF-shRNA activity**

THP-1 cells were transformed with 1μg plasmid encoding shRNA constructs specific for mouse TNF-α (Ambion) by nucleofection. Following overnight culture to allow degradation of TNF-α mRNA, 1 μg/mL LPS was added to the cultures in order to stimulate the secretion of TNF-α. Cytokine concentration in the supernatant was determined by ELISA after 24 or 48 hours.

**Antibody controls**

294T, MCF7, Mel-28, and BT-20 cell lines were plated overnight in 96 well plates. αIFN-γ **(**MAB285) or αTNF-α (Mab1) was added at 10 μg/mL followed by a titration of recombinant human IFN-γ (A & B) or TNF-α (C & D). After 3-4 days, cultures were pulsed with 0.5 μCi H^3^-thymidine overnight to measure the viability of the cell populations.

**Mycobacterial inhibition assay**

BCG-infected macrophages and γ_9_δ_2_ T cells were prepared as in the main text. At the start of the co-culture period, the cell-permeable general caspase inhibitor, zVAD.fmk (10 μM), or autophagy inhibitors, 3-methyadenine (5 mM) or Wortmannin (10 μM), were added. After 3 days of culture, the viability of intracellular mycobacteria was quantitated by H^3^-uridine incorporation.

**IFN-β and NO analysis**

3 days after co-culture of BCG-infected macrophages alone or co-cultured with protective γ_9_δ_2_ T cells, supernatants were removed for further analysis. IFN-β was measured in culture supernatants by ELISA (Interferon) while nitric oxide was measured by Griess reaction.

**Flow cytometry**

To validate the effectiveness of the granzyme A-targeting siRNA, transduced and non-transduced γ_9_δ_2_ T cells were stained with fluorescent antibodies directed against extracellular markers followed by fixation and permeabilization (Cytofix/CytoPerm, BD Bioscience) and intracellular staining for granzyme A.
